# Pronounced Plasticity Caused by Phase Separation and β-relaxation Synergistically in Zr–Cu–Al–Mo Bulk Metallic Glasses

**DOI:** 10.1038/s41598-017-01283-5

**Published:** 2017-04-27

**Authors:** Tuo Wang, Lu Wang, Qinjia Wang, Yanhui Liu, Xidong Hui

**Affiliations:** 1 State Key Laboratory for Advanced Metals and Materials, University of Science and Technology Beijing, Beijing, 100083 China; 2 Institute of Physics, Chinese Academy of Sciences, Beijing, 100190 China

## Abstract

Bulk metallic glasses (BMGs) are known to have extraordinary merits such as ultrahigh strength and dynamic toughness *etc*. but tied to the detrimental brittleness, which has become a critical issue to the engineering application and understanding the glass nature. In this article, we report a new class of Zr-Cu-Al-Mo BMGs with extraordinary plastic strain above 20%. “Work-hardening” effect after yielding in a wide range of plastic deformation process has been detected for this kind of BMGs. Compositional heterogeneity, which can be classified into ZrMo- and Cu-rich zones, was differentiated in this kind of BMG. Pronounced humps have been observed on the high frequency kinetic spectrum in Mo containing BMGs, which is the indicator of β-relaxation transition. The underlying mechanism for the excellent plastic deforming ability of this class of BMGs is ascribed to the synergistic effects of soft ZrMo-rich glass formed through phase separation and abundant flow units which related to β-relaxation.

## Introduction

Bulk metallic glasses (BMGs) are formed when the crystallization of supercooling liquids is suppressed during the cooling process^1–6^. The grain boundary free structure with disordering atomic packing in long range renders BMGs high strength and elasticity, excellent corrosion resistant and soft magnetic properties *etc*., but brings about harmful brittleness in a majority of BMGs. Especially, the ductility of some BMGs even approaches to zero^7–9^, which may result in catastrophic failure of the BMGs when they are imposed a stress larger than the yielding strength. Therefore, the low plasticity or severe brittleness has become a critical issue to the application of BMGs for structural materials. Fundamentally, the lack of plasticity is also detrimental to understanding the glass nature, such as the glass transition and deformation mechanism of BMGs.

Unlike crystalline solids which are homogeneously deformed through the gliding and proliferation of dislocations, BMGs are plastically deformed at room temperature in the frame of shear transformation zones (STZs) theory, which was proposed by Argon^10^. That is to say, the STZs can be regarded as a kind of reversible flow unit for deformations so that the plastic deformation of BMGs is only confined in shear bands (SBs) at room temperature. Thus the strategy to improve the plasticity of BMGs has being focused on activating more SBs and hindering the propagation of SBs. Based on this cognation, a series of pathways, *e*. *g*. alloying optimization by the elements with large Poisson’s ratio^11^, *in-situ* crystallization of ductile phase in BMG matrix^12, 13^, formation of nanometer scaled structural chemical heterogeneity^14^, increasing the free volume^15, 16^ *etc*. have been attempted in recent years. Beside above methods, composition design by including the elements with positive mixing enthalpy (ΔH_x_) has been also proved an effective way to improve the plasticity of BMGs^17–20^. For instance, the compressive plasticity of Cu_46_Zr_47_Al_7_ BMG^17^ can be increased from 0.59% to 7.47% when Cu is substituted partly by 1% Fe (the ΔH_x_ between Fe and Cu is ΔH_Fe−Cu_ = +13 kJ/mol^21^). So far as present, Fe, Nb, Ag and Pd^17, 22, 23^ elements *etc*. have been used as the plasticizers for Zr-, Cu- and Ti-based BMGs.

Johari-Goldstein type of relaxation (β-relaxation) is an intrinsic and universal feature of supercooled liquids and glasses, and the basic physics of β-relaxation is of practical significance to many features and properties of glasses^24–26^. So far as present, β-relaxation, which manifests as distinct peak or excess wing on the α-relaxation peak of the dynamic mechanical analysis (DMA) spectrum, has been found in La-, Sm-, Ce-, Pd-, Pt-, Cu-, and Zr-based BMGs. And it has also shown that most of these BMGs have pronounced plastic deforming ability. Especially, Yu *et al*.^27^ have reported that pronounced macroscopic tensile plasticity has been achieved in a La-based metallic glass which possesses strong β relaxation. It has been proved that the activation energy of β-relaxation and the potential-energy barriers of STZs in MGs are nearly equivalent^25, 28^. This means that the localized atomic motion responsible for β-relaxation in a glassy state may become the principal resource of “potential STZs” when α relaxation is frozen below glass transition temperature, T_g_^29^. In this sense, the underlying mechanism of plastic deformation of BMGs can be also understood from the point of view of β-relaxation.

In this work, we report a new class of Zr-Cu-Al-Mo BMGs which show extraordinary plasticity and “work hardening”. The plasticity in BMGs has been previously interpreted either in terms of phase separation or β-relaxation theory. Nevertheless, it is scarce that both the phase separation and β-relaxation are simultaneously observed in a BMG. Here we show that there exists both phase separation and β-relaxation in Zr-Cu-Al-Mo BMGs, which endow these BMGs with outstanding plastic deformability. We argue that the underlying mechanism for the extraordinary deformability of BMGs intrinsically stems from these two kinds of physical phenomena.

## Results

**Figure 1:**
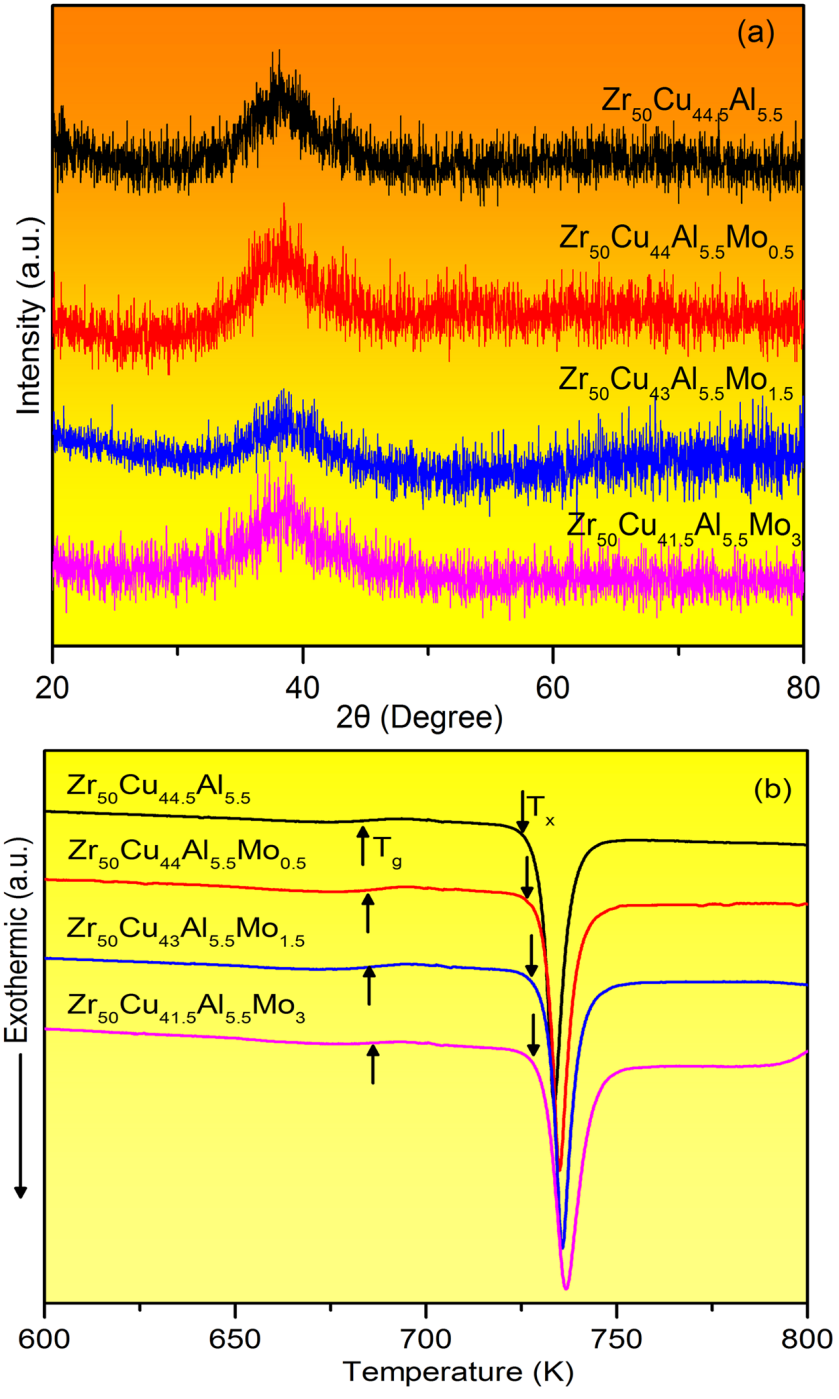
(**a**) XRD patterns and (**b**) DSC curves of Zr_50_Cu_44.5−x_Al_5.5_Mo_x_ (x = 0, 0.5, 1.5, 3 at.%) glassy rods with the diameter of 2 mm.

The glassy structure of as-cast Zr_50_Cu_44.5−*x*_Al_5.5_Mo_*x*_ (*x* = 0, 0.5, 1.5, 3 at.%, denoted as ZCA, ZCAM0.5, ZCAM1.5, ZCAM3, respectively, in the following context) was characterized in the first place by XRD and DSC methods. The XRD patterns of these specimens are shown in Fig. 1(a). There is only a broad halo peak on each curve of the as-cast alloys and no other diffraction peak of crystalline phases can be observed, indicating that glassy structure was formed in all these as-cast alloys. Figure 1(b) illustrates the DSC curves tested at the heating rate of 0.083 K/s for the as-cast glassy rods. Each curve exhibits an obvious glass transition, followed by an extended supercooling liquid region and a crystallization process. It is seen that with the increase of Mo content, both the glass transition temperature, T_g_, and the onset crystallization temperature, T_x_, are slightly increased.

**Figure 2:**
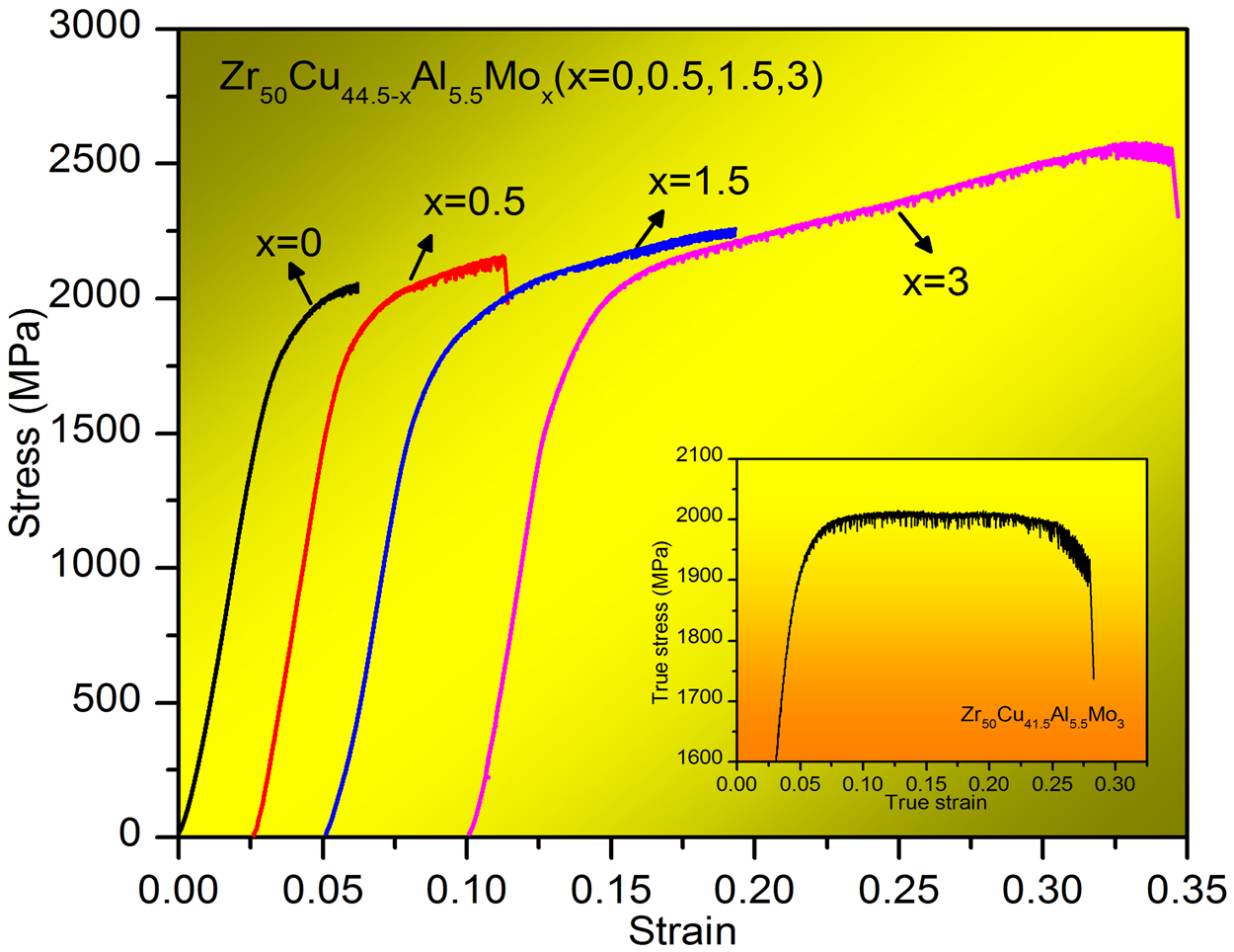
Compressive engineering stress-strain curves of as-cast samples Zr_50_Cu_44.5−*x*_Al_5.5_Mo_*x*_ (*x* = 0, 0.5, 1.5, 3 at.%). The inset shows the true stress-strain curve of the sample with *x* = 3.

Figure 2 shows the compressive stress-strain curves for the four BMG rods with a diameter of 2 mm. Like other typical BMGs, these BMG alloys exhibit engineering elastic strain limits of ~2%. However, it is found that the addition of Mo results in a decrease in yielding strength from 1680 MPa to 1590 MPa for ZCA and ZCAM3, respectively. In contrast, the fracture compressive strengths, *σ*_c_, and plastic strains, *ε*, of these BMGs are greatly enhanced. The *σ*_c_ is increased from 2055 MPa to 2579 MPa, and *ε* from 2.6% to 20.4% for ZCA and ZCAM3, respectively. The true stress-strain curve of ZCAM3 is also presented in the inset of Fig. 2. It is noticeable that the flow stress after yielding is significantly increased by 33.2% with further increase of plastic strain up to 13.6% after yielding, *i*.*e*., exhibiting a so-called distinct “work-hardening” behavior. This kind of “work-hardening” phenomenon has been found in Cu_47.5_Zr_47.5_Al_5_ by Das *et al*.^30^ in 2005. They reported that the strength and strain of Cu_47.5_Zr_47.5_Al_5_ BMG reach up to 2265 MPa and 18%, respectively. The “work-hardening” capability and ductility are attributed to a unique structure correlated with atomic-scale inhomogeneity.

**Figure 3:**
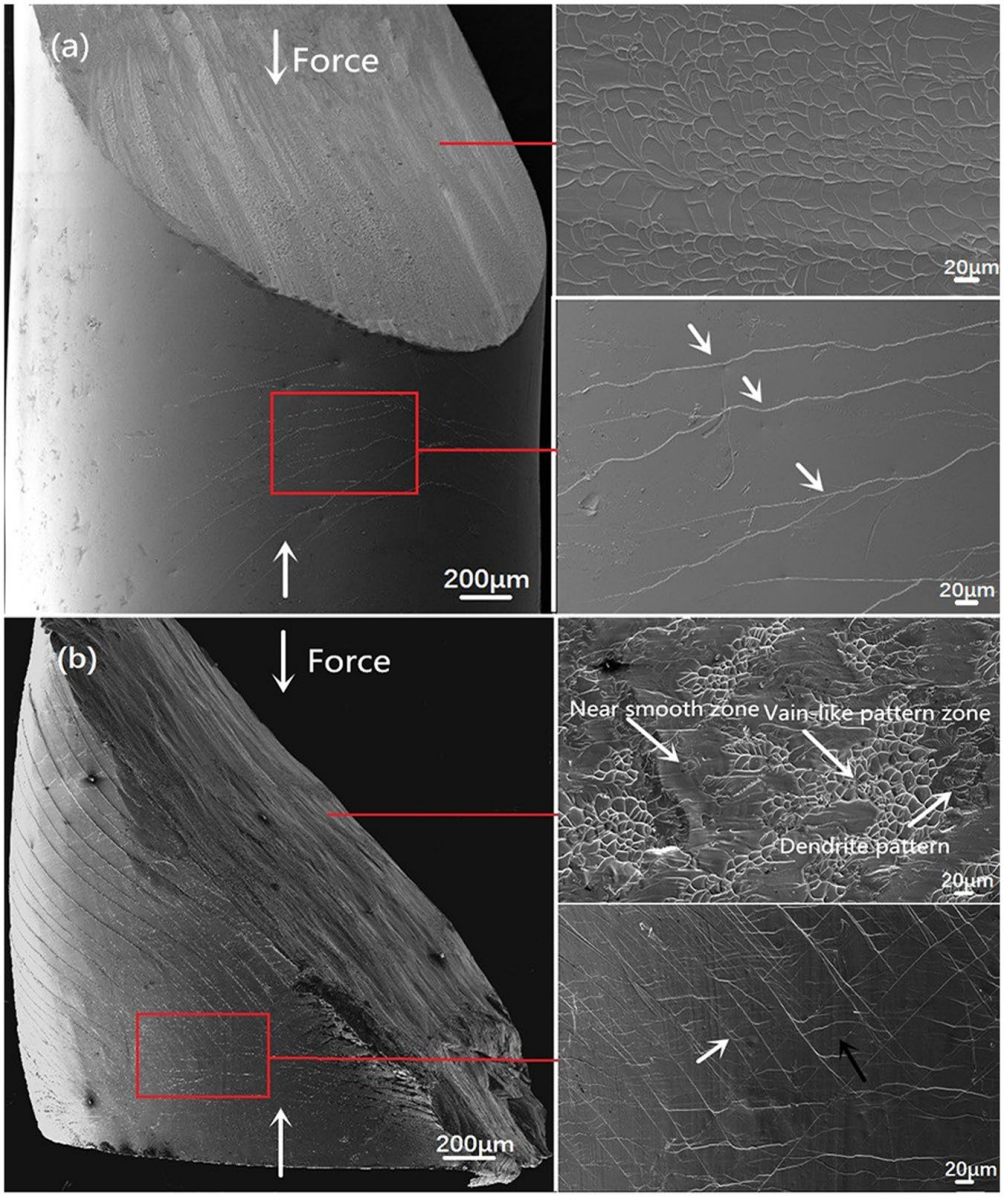
SEM images of the fracture and lateral surfaces of deformed (**a**) Zr_50_Cu_44.5_Al_5.5_, and (**b**) Zr_50_Cu_41.5_Al_5.5_Mo_3_ BMG rods, respectively.

The fracture and lateral surfaces of the deformed ZCA and ZCAM3 BMGs are shown in Fig. 3. For the ternary ZCA BMG (Fig. 3(a)), the specimen still holds the cylindrical geometry after fractured, indicating the poor plasticity for this BMG. The compressive fracture angle, *θ*, between the loading axis and the fracture plane is smaller than 45°, which is similar to that observed in most of BMGs^31^. Relatively homogeneous vein-like patterns are distributed on the fracture surface, meaning that severe local plastic deformation took place in SBs before the specimen was fractured. At a higher magnification, the SBs are visible on the lateral surface of specimen. Primary SBs are distributed with the average spacing of about 100 μm. Slight branching from these primary SBs are also observed, but little secondary SBs were formed. For the quaternary ZCAM3, however, the cylindrical shape of the specimen was change to barrel shape geometry, indicating that this BMG has gone through a high plastic deformation. The minimum spacing of primary SBs was estimated to be lower than 10 μm. Interaction and intersection of primary (pointed by white arrows) and secondary (pointed by﻿ black arrows) SBs can be observed on the surface of specimen, resulting in the formation of secondary SBs. These secondary SBs move in rather wavy route and even cut through several primary SBs. The formation of a high density of primary and secondary SBs in this novel BMG decreases the propagation velocity of SBs and restricts the catastrophic failure. Moreover, it is also found that the fracture surface of ZCAM3 BMG exhibits high localized morphology. Two types of deformation zones, *i*.*e*., vein-like pattern and near smooth zone, were formed on the fracture surface. And the size of these vein patterns of ZCAM3 is quite smaller than that of ZCA BMG. At the boundary between these two zones, fine dendrite pattern can be also observed. All the above results of SBs and fracture morphologies demonstrate that the plastic deformation of ZCAM3 BMG has been accomplished through highly localized shear banding.

**Figure 4:**
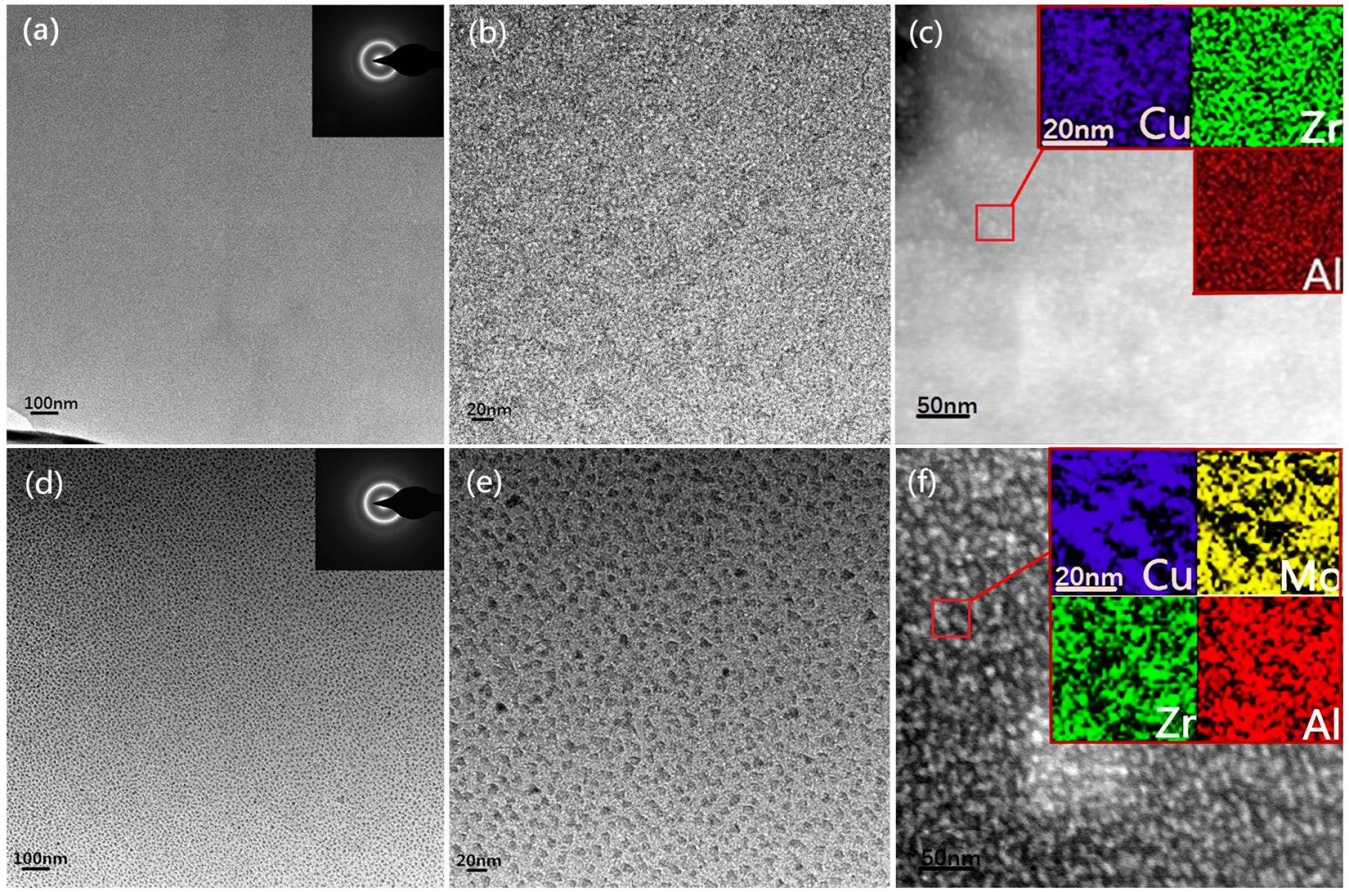
(**a**) and (**d**) low magnification bright-field TEM images and the corresponding SAED patterns, (**b**) and (**e**) high magnification bright-field TEM images, (**c**) and (**f**) STEM images and the elemental distribution mapping of the regions in the boxes for Zr_50_Cu_44.5_Al_5.5_ and Zr_50_Cu_41.5_Al_5.5_Mo_3_ samples, respectively.

TEM study on the structure and heterogeneity for these BMGs was conducted in this work. As shown in Fig. 4(a) and (b), the low and high magnification of TEM images of as-cast ZCA BMG show no contrast in the image. The corresponding selected area electron diffraction (SAED) pattern as shown in the inset of Fig. 4(a) indicates that there is only a diffusive halo, confirming the glassy nature of this BMG. The STEM image and the elemental distribution mapping (Fig. 4(c)) for ZCA indicate that the composition distribution in this BMG is almost homogeneous. However, the structural feature and compositional distribution of ZCAM3 BMG are different from those of ZCA BMG. As shown in the inset of Fig. 4(d), the SAED pattern does not show any diffraction spots, implying that the alloy is of glassy structure and it is found that there are two different kinds of interconnected networks with bright and dark contrasts, strong contrast fringes can be obsereved﻿ in the high magnification of TEM image as shown in Fig. 4(e). The size of the network is about 10–20 nm. By the EDX analysis, the nominal composition of bright zone is estimated to be Zr_52.76_Cu_37.61_Al_3.56_Mo_6.07_, and the dark zone is Zr_46.29_Cu_48.82_Al_3.00_Mo_1.89_. Figure 4(f) shows the elemental distribution mapping for ZCAM3 BMG. It is seen that all the distributions of Zr, Cu, Al and Mo in Fig. 4(f) are more heterogeneous than those in Fig. 4(c). In addition, it seems that Zr and Mo are diffused in the region depleted with Cu in Fig. 4(f). Based on the results of EDX and elemental distribution mapping analysis, it can be deduced that the bright zone is ZrMo-rich, and the dark zone is Cu-rich glass in the Fig. 4(e).

**Figure 5:**
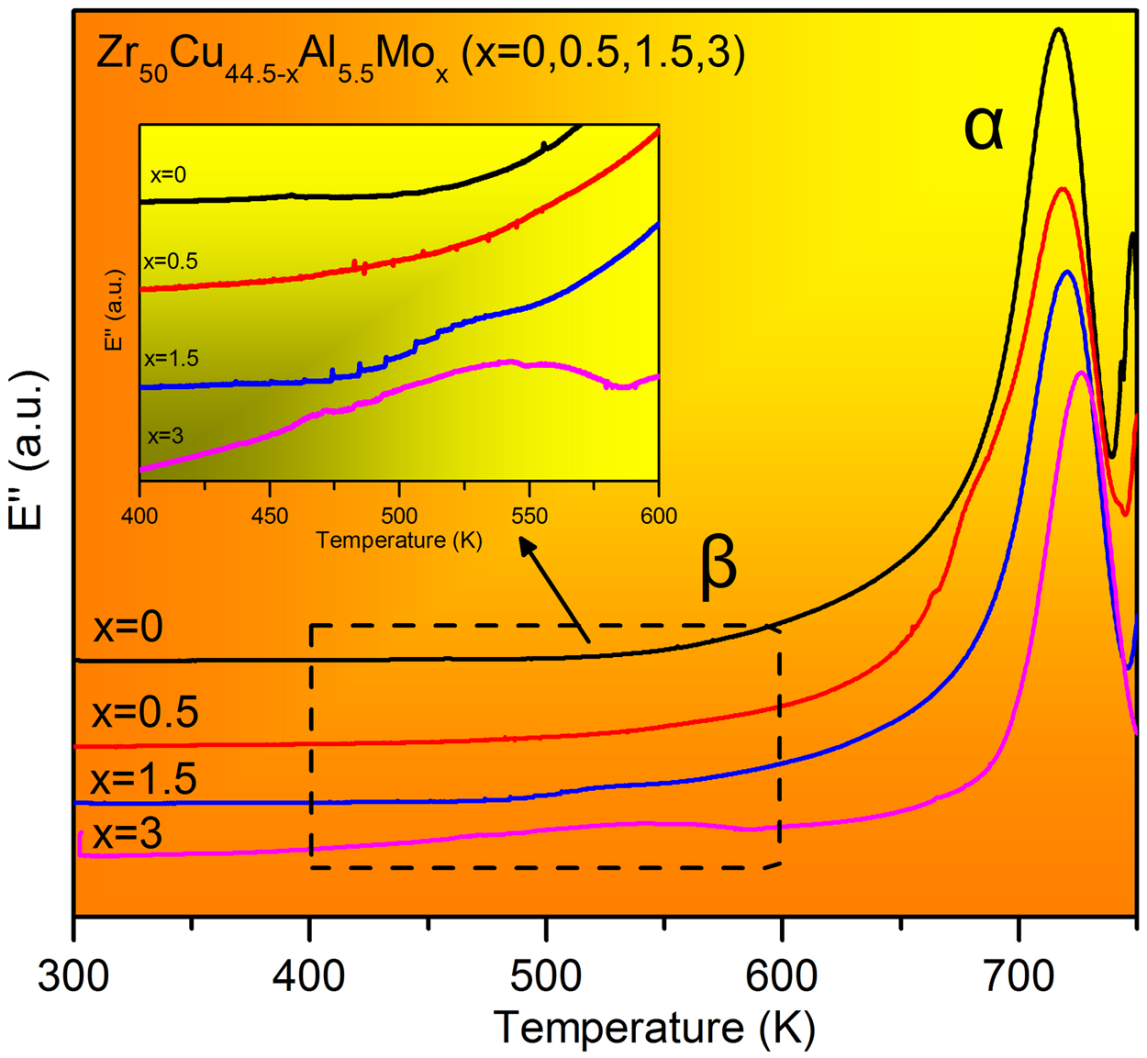
DMA curves of Zr_50_Cu_44.5−x_Al_5.5_Mo_x_ (x = 0, 0.5, 1.5, 3 at.%) BMGs, the inset is the enlarged image measured from 400 K to 600 K.

DMA measurement is an effective method in detecting the mechanical relaxations in glassy solids and supercooled liquids^25, 27, 28^. Figure 5 shows the loss modulus E” (in order to show a clear difference between these samples at 400 K to 600 K, the E” was given in the relative value) as function of temperature under the testing frequency *f* = 1 Hz for these BMGs. Besides α-relaxation peaks around T_g_, one can see that β-relaxation occurs in the temperature range of about 0.8 T_g_. However, the degree of β-relaxation of the BMGs is different. As shown in Fig. 5, β-relaxation appears to be absent for ZCA BMG. The feature of β-relaxation in ZCAM0.5 BMG exhibits an excess tail around 0.8 T_g_, whereas ZCAM1.5 and ZCAM3 BMGs show pronounced humps for the β-relaxation controls. And compared with ZCAM1.5 BMG, ZCAM3 BMG has a wider and more obvious hump.

## Discussion

The above results have demonstrated that enhanced plasticity can be achieved in Zr-Cu-Al-Mo BMGs through the manipulation of composition. Here, we attempt to explain the intrinsic origin of the extraordinary plasticity in this kind of BMGs from the view point of phase separation and relaxation process.

From Fig. 4(c) and (f), it is seen that the elemental distribution of ZCAM3 BMG is more heterogeneous than that of ZCA BMG. And the morphological feature of ZCAM3 BMG is very similar to that formed by the liquid spinodal decomposition, which has been put forward by Cahn in 1961^32^. The results of EDX and elemental distribution mapping have proved the chemical heterogeneity in these BMGs. It is deduced that the chemical inhomogeneity can be ascribed to the liquid spinodal decomposition. The formation of heterogeneity in ZCAM3 BMG can be also interpreted from the heat of mixing among the components. It is known that there is a positive heat of mixing between Mo and Cu (ΔH_Cu−Mo_ = 19 kJ/mol^21^). Thus Cu and Mo atoms may separate each other during the solidification, resulting in the formation of Cu-rich or Mo-rich zone. On the other hand, due to the affinity between Mo and Zr (ΔH_Zr−Mo_ = −6 kJ/mol^21^), Mo atoms can be trapped by Zr atoms, and thus their mobilities are decreased, causing the formation of ZrMo-rich zone. It is also noted that the diffusion coefficient of Cu in Zr-Cu alloy is 10^5^ times larger than that of Zr^33^. Kinetically, it is reasonable to deduce that Cu-rich clusters can be more easily dispersed in undercooled liquid, and the mobility of these clusters may be suppressed by Zr and Al atoms which have larger atomic radii^21^ than Cu atoms. Therefore, it is the phase separation in ZCAM3 BMG which results in more heterogeneous structure than that in ZCA BMG.

Then, what’s the mechanism for the pronounced plasticity of ZCAM3 in this work? Here we argue that the underlying intrinsic origin for this phenomenon is the β relaxation caused by the formation of Cu-rich and ZrMo-rich zone. To interpret this this mechanism, we take into account of the fluctuations of chemical interactions for these alloys. The mean chemical affinities, ΔH, for these MGs are estimated by ${\rm{\Delta }}H=4\sum _{A\ne B}{H}_{AB}{c}_{A}{c}_{B}$ as suggested by Takeuchi *et al*.^21^, where H_AB_ is the mixing of enthalpy between A and B atoms, *c*_A_ and *c*_B_ are the atomic percent in the system. It can be calculated that ΔH_ZCA_ is −25.41 kJ/mol and ΔH_ZCAM3_ is −23.47 kJ/mol. In Yu’s work^34^, they have found that Al-free Cu_50_Zr_50_ MG manifests β-relaxation event, whereas the Al alloying drastically suppresses this fast relaxation. It is easily to know that the ΔH for Cu_50_Zr_50_ MG is −23 kJ/mol, which is quite close to that of ZCAM3 BMG.

The overall enthalpy of mixing is a factor to influence the β-relaxation event. But the comparable magnitudes of interactions among all the constituting atoms is another critical factor to determine the occurrence of β-relaxation event. As shown in the TEM and EDX results (Fig. 4), the addition of Mo results in the formation of two kinds of regions with the compositions of Zr_52.76_Cu_37.61_Al_3.56_Mo_6.07_ and Zr_46.29_Cu_48.82_Al_3.00_Mo_1.89_ in ZCAM3 BMG. The ΔHs for the ZrMo-rich and Cu-rich regions are calculated to be −21.41 kJ/mol and −22.81 kJ/mol, respectively. These data mean that the phase separation leads to more comparable chemical interaction in ZrMo-rich and Cu-rich zones. In Cu-rich zone, more Zr-Cu pairs form to compete with Zr-Al pairs. In ZrMo-rich zone, the depletion of Cu and the rich of Mo make chemical interactions more balanced, *i*.*e*., the Zr-Mo and Al-Mo atomic pairs are increased but the Zr-Cu pairs reduced. According to Stevenson and Yu’s theoretical and experimental work^34^, string-like configuration with comparable interactions among the constituents is beneficial to the occurrence of β-relaxation. In this sense, β-relaxation seems to take place more easily in the phase separated ZrMo-rich than that in Cu-rich zone.

**Figure 6:**
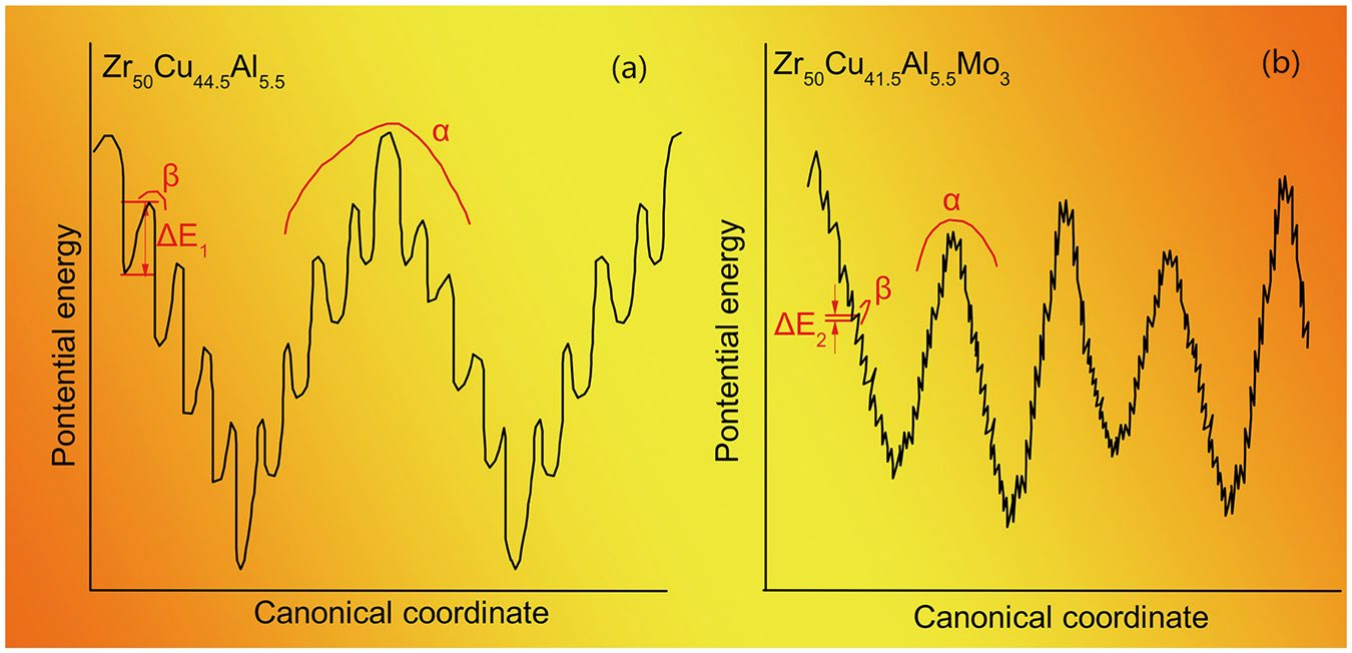
Schematic diagrams of the potential energy landscape for Zr_50_Cu_44.5_Al_5.5_ and Zr_50_Cu_41.5_Al_5.5_Mo_3_ BMGs.

From the potential energy landscape perspective developed by Stillinger^35, 36^, Wales^37^, and Sastry *et al*.,^38, 39^ the β-transitions can be identified as stochastically activated hopping events across “subbasins” confined within the inherent “megabasin” (intrabasin hopping, which is corresponding α-relaxation). In this work, the difference between the modes of β-relaxation event can be illustrated by a schematic diagram shown in Fig. 6. The ZCA BMG can be regarded as being homogeneous with strong interaction (Fig. 6(a)). So there is only one kind of inherent intrabasins with deep valleys, which represents the activation energy of α-relaxation. Within the megabasins, there are relatively small subbasins, which represents the energy barriers (ΔE_1_) for β-relaxation events. Due to the strong interaction among atomic pairs, the activation energies for β-relaxation are relatively large. As for ZCAM3, the phase separation in ZCAM3 results in more complex inherent intrabasins. Compared with the intrabasins of ZCA BMG, the valleys of the intrabasins for ZCAM3 BMG may be shallower, considering that the chemical interaction in both Cu-rich and ZrMo-rich zones are weaker than that in ZCA BMG (Fig. 6(b)). Moreover, in the intrabasins of these two compositional zones, there are abundant subbasins, of which the activation energies (ΔE_2_) (Fig. S1) are much lower than those in ZCA BMG and mean that the β-relaxation took place easily in ZCAM3 BMG.

In light of above scenario, the structural origin for the extraordinary plasticity of Zr-Cu-Al-Mo BMGs can be well understood. In ZCAM3 BMG, the addition of Mo results in the formation of ZrMo-rich and Cu-rich zones through phase separation. β-relaxation takes place in the ZrMo-rich zone due to the low activation energies for the β-relaxation of “string-like configuration” as proposed by Stevenson *et al*.^40^. The reversible back-and-forth movement of the chain of atoms in ZrMo-rich zone may trigger another kind of β-relaxation in Cu-rich zone. Since the activation energies of β-relaxation are equivalent to the energy barriers of STZs, the β-relaxation event will lead to the initiate of STZs. In other words, a large scale of β-relaxation events are actually the indicators of abundant flow units^27, 41^ in a BMG, and these flow units can be regarded as the liquid-like zones in BMGs. According to the STZ theory, the plastic flow of metallic glasses will proceed through the formation and cooperative shearing of unstable STZs.

Of the ZrMo-rich and Cu-rich zones, we argue that ZrMo-rich zone is a soft glass considering that the chemical interaction is weaker in this zone than that in Cu-rich zone. Thus the critical shear stress (CSS) of yielding is lower in this zone than that in Cu-rich zone. Then primary STZs will be initiated in the soft ZrMo-rich zone. Macroscopically, these isolated STZs can be considered to distribute uniformly in the BMG. With further increase of external stress, the primary STZs will propagate in light of a certain mechanics criterion until they encounter the hard Cu-rich zone. Due to the hindering effect of Cu-rich zone to propagation of STZs, the primary STZs have to be branched or thickened, resulting in the multiplication of SBs. In this process, the internal stress will increase with the increase of plastic strain, which is so called “work hardening”. With further increase of applied stress to the CSS of Cu-rich zone, STZs will be motivated in this kind of hard glass. The shear deformation may spread over to the whole specimen. In the last stage of plastic deformation, the propagation of STZs will be accelerated and the energy in STZs accumulated continuously, resulting in the increase of temperature in STZs. Finally, the plastic deformation of BMGs reaches softening process, and causes catastrophic fracture.

## Conclusions

In summary, (1) this work presents a new class of Zr-Cu-Al-Mo BMGs with enhanced engineering compression fracture strength up to 2579 MPa and plastic strain above 20%. This kind of BMG exhibits obvious “work-hardening” behavior after yielding in a wide range of plastic deformation process. (2) High density of primary SBs with minimum spacing lower than 10 μm and incrossed wavy secondary SBs have been formed in the novel Zr-Cu-Al-Mo BMGs. The fracture surface of ZCAM3 exhibits high localized morphology composed of vein-like patterns and near smooth zones. (3) It has been confirmed that the distributions of Zr, Cu, Al and Mo in the novel Zr-Cu-Al-Mo BMG are strongly heterogeneous. ZrMo- and Cu-rich glass can be differentiated, which were formed through liquid spinodal decomposition. (4) Obvious β-relaxation can be detected in ZCAM3, whereas an excess tail on the α-peak is observed in the DMA of ZCA. (5) The underlying mechanism for the extraordinary plastic deforming ability of this class of BMGs can be attributed to the formation of soft ZrMo-rich glass through phase separation and the existence of abundant flow units which related to β-relaxation.

## Methods

The master alloy ingots of Zr_50_Cu_44.5−*x*_Al_5.5_Mo_*x*_ (*x* = 0, 0.5, 1.5, 3 at.%) were prepared by using pure Zr, Cu, Al and Mo metals with a purity of above 99.5 mass % in an arc-melting furnace in a Ti-gettered argon atmosphere. To ensure compositional homogeneity, each ingot was remelted at least five times. Subsequently, cylindrical samples with 2 mm in diameter and 70 mm in length were prepared by suction casting in a copper mold. TEM specimens were ion-milled using a Gatan 691 Precision Ion Polishing System with liquid-nitrogen cooling. The uniaxial compression tests were performed with a CMT4305 universal testing machine at room temperature with a strain rate of 2 × 10^−4^ s^−1^. The compressive specimens with 2 mm in diameter and 4 mm in height were cut from these as-cat rods, and the ends were polished carefully to ensure parallelism. At least three specimens were measured for each composition to ensure that the results were reproducible. The true strain was calculated as *ε*_true_ = ln(1 + *ε*) and the true stress as σ_true_ = σ(1 + *ε*). The lateral surfaces and fracture surfaces after failure were investigated by scanning electron microscopy (SEM) using an SUPRA55 FE-SEM. The glassy nature of the alloy rods were confirmed by X-ray diffractometer (XRD) using a Brook D8 ADVANCE (Cu Kα radiation). The crystallization kinetics of BMG samples was investigated by Netzsch STA 449C Differential scanning calorimetry (DSC) under an argon atmosphere at the 5 K/min heating rates. Further microstructural investigations were examined by a high-resolution transmission electron microscopy (HRTEM, Tecnai G2 F20) with energy dispersive X-ray spectrometers (EDX). ﻿The dynamical mechanical analysis was performed with TA Q800 type of machine at a heating rate of 5 K/min and a frequency of 1 Hz. The samples with dimensions of about 35 mm × 1 mm × 1 mm were prepared using a wire-electrode cutting for DMA test.

## Electronic supplementary material

Supplementary Information
