# Supplementary material for: Pronounced Plasticity Caused by Phase Separation and β-relaxation Synergistically in Zr–Cu–Al–Mo Bulk Metallic Glasses
Source: Sci Rep. 2017 Apr 27;7:1238. doi: 10.1038/s41598-017-01283-5 (PMC5430678; doi:10.1038/s41598-017-01283-5)
Supplement: Supplementary file 1 — Supplementary Information [file 41598_2017_1283_MOESM1_ESM.pdf]

Supplementary Information

Pronounced Plasticity Caused by Phase Separation and β-relaxation Synergistically in Zr–Cu–Al–Mo Bulk Metallic Glasses

T. Wang,[a] L. Wang,[a] Q. J. Wang,[a] Y.H. Liu,[b] X.D. Hui[a,*]

[a]State Key Laboratory for Advanced Metals and Materials,
University of Science and Technology Beijing, Beijing 100083, China
[b]Institute of Physics, Chinese Academy of Sciences, Beijing 100190, China

Supplementary Figures

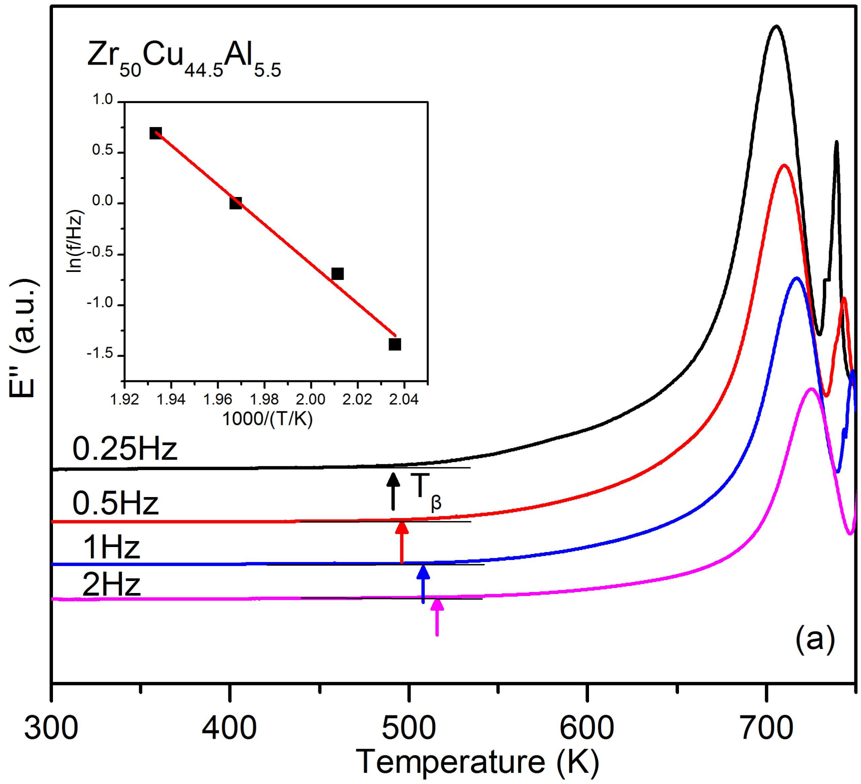

*Corresponding authors. E-mail: xdhui@ustb.edu.cn (X. D. Hui).

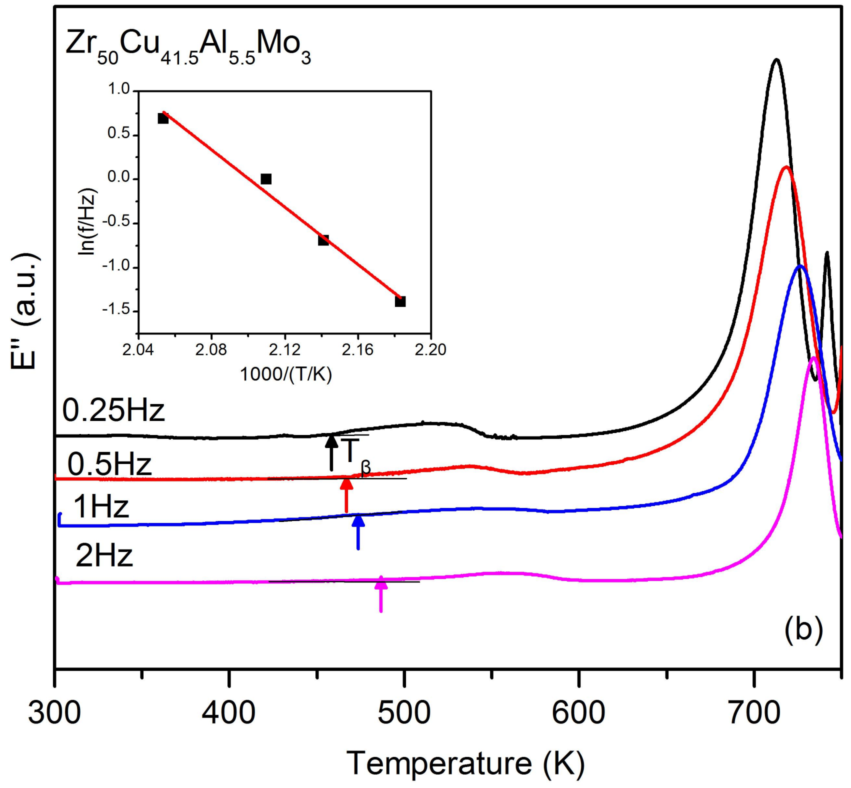

Fig. S1 The temperature dependence on the loss modulus *E"* measured with frequency *f* , the inset plots are *ln(f)* vs the onset temperature of β-relaxation (T_β) for the alloys: (a) $Zr_{50}Cu_{44.5}Al_{5.5}$ (b) $Zr_{50}Cu_{41.5}Al_{5.5}Mo_3$

Supplementary Note

The temperature dependence on the *E"* as a function of frequency (*f*) is shown in Fig. S1. The inset of Fig. S1 shows the *f* dependence on the onset temperature of β-relaxation (T_β) which is marked on the DMA curver. These points were fitted by the Arrhenius equation

$$f = f_\infty \exp(-E_\beta / RT) \tag{1}$$

Where f_∞ is the prefactor, E_β is the activation energy of β-relaxation, *R* is the ideal gas constant and *T* is the temperature. The Eq. (1) can be also written as

$$\ln(f) = \ln(f_\infty) - E_\beta / RT \tag{2}$$

So, $-E_\beta/R$ is the slope of the line on a plot of ln(*f*) versus $1/T_\beta$.

The E_β is determined to be 162 ± 12kJ/mol and 135 ± 11kJ/mol for $Zr_{50}Cu_{44.5}Al_{5.5}$ (ZCA) and $Zr_{50}Cu_{41.5}Al_{5.5}Mo_3$ (ZCAM3) from the Arrhenius plots, resepectively. And these values are corresponding to ΔE_1 and ΔE_2, resepectively.
